# Impact of Opioid Use on Duration of Therapy and Overall Survival for Patients with Advanced Non-Small Cell Lung Cancer Treated with Immune Checkpoint Inhibitors

**DOI:** 10.3390/curroncol31010017

**Published:** 2024-01-03

**Authors:** Philip Young, Omar Elghawy, Joseph Mock, Emmett Wynter, Ryan D. Gentzler, Linda W. Martin, Wendy Novicoff, Richard Hall

**Affiliations:** 1 Department of Medicine, Division of Hematology/Oncology, University of Virginia, Charlottesville, VA 22903, USA; pry4dm@uvahealth.org (P.Y.); rg2uc@uvahealth.org (R.D.G.); 2 School of Medicine, University of Virginia, Charlottesville, VA 22903, USA; omar.elghawy@pennmedicine.upenn.edu; 3 Department of Medicine, University of Virginia, Charlottesville, VA 22903, USA; 4 Department of Surgery, Division of Thoracic and Cardiovascular Surgery, University of Virginia, Charlottesville, VA 22903, USA; 5 Department of Public Health Sciences and Orthopedic Surgery, University of Virginia, Charlottesville, VA 22903, USA

**Keywords:** non-small cell lung cancer, immune checkpoint inhibitors, opioids, duration of therapy, overall survival

## Abstract

Immune checkpoint inhibitors (ICI) have significantly improved outcomes in advanced non-small cell lung cancer (NSCLC). We evaluated the effect of opioid use on outcomes in patients receiving ICI either alone or with chemotherapy. We conducted a retrospective review of 209 patients with advanced NSCLC who received an ICI at the University of Virginia between 1 February 2015 and 1 January 2020. We performed univariate and multivariate analyses to evaluate the impact of opioid use on duration of therapy (DOT) and overall survival (OS). Patients with no or low opioid use (n = 172) had a median DOT of 12.2 months (95% CI: 6.9–17.4) compared to 1.9 months (95% CI: 1.8–2.0) for those with high opioid use (n = 37, HR 0.26 95% CI: 0.17–0.40, *p* < 0.001). Patients with no or low opioid use had a median OS of 22.6 months (95% CI: 14.8–30.4) compared to 3.8 months (95% CI: 2.7–4.9) for those with high opioid use (HR 0.26 95% CI: 0.17–0.40 *p* < 0.001). High opioid use was associated with a shorter DOT and worse OS. This difference remained significant when accounting for possible confounding variables. These data warrant investigation of possible mechanistic interactions between opioids, tumor progression, and ICIs, as well as prospective evaluation of opioid-sparing pain management strategies, where possible.

## 1. Introduction

Immune checkpoint inhibitors (ICIs) have changed the treatment landscape of non-small cell lung cancer (NSCLC) and led to improvements in overall survival (OS) and progression-free survival (PFS) over chemotherapy alone [1,2,3,4,5,6,7]. ICIs can lead to prolonged survival, with updated analysis from the KEYNOTE-024 trial showing a 5-year overall survival of 31.9% among patients with treatment-naïve NSCLC and PD-L1 ≥ 50% treated with pembrolizumab [8]. Despite these encouraging results, only a minority of patients receiving these therapies achieve a long-term response, and median survival for metastatic disease remains at 22–26 months, depending on the treatment and patient population [8,9,10]. Understanding the factors that influence which patients respond to ICIs has become an important area of clinical research.

The mechanism by which immune checkpoint inhibitors (anti-CTLA-4 and anti-PD-1/PD-L1) induce tumor rejection is thought to rely on the activation of T-cell activity. CTLA-4 and PD-1 surface T-cell receptors attenuate T-cell activity upon interaction with their corresponding ligands (B7-1/B7-2 and PD-L1/PD-L2, respectively) and are a mechanism by which tumors induce tolerance and evade killing by cytotoxic T-cells [11]. Immune checkpoint inhibition induces tumor rejection by blocking this interaction, leading to the reinvigoration of cytotoxic T-cells, the expansion of specific tumor-infiltrating T-cell populations, and the depletion of regulatory T-cells [11,12].

Opioids, particularly morphine, modulate T-cell activity and T-regulatory cell function and thus may interfere with response to immunotherapy. T-lymphocytes express opioid receptors, and chronic opioid use increases opioid receptor expression [13]. In vitro studies indicate that morphine leads to decreased T-cell activity through the inhibition of IL-2 transcription and T-cell receptor (TCR) signaling [13]. Morphine and fentanyl exposure, at 12 weeks and 7 days, respectively, increases the number of regulatory T-cells (Tregs) [13]. In addition to the direct effect of morphine on T-cell activity, chronic opioid use has been associated with decreased T-cell function and reduced NK cell cytotoxicity by stimulation of the hypothalamic–pituitary adrenal axis and subsequent release of endogenous glucocorticoids [14].

Opioids also compromise the gut barrier and alter the gut microbiome in mouse models [15,16]. Alterations in the gut microbiome following antibiotic use has been shown to mediate resistance to ICIs [17,18]. In humans, opioid use is associated with an alteration of gut microbiota in patients with type 2 diabetes [19] as well as those with opioid use disorder [20].

Given these putative mechanisms by which opioid use may impair the immune response, along with the common use of opioids to treat cancer-related pain among NSCLC patients, we sought to investigate whether concomitant opioid use was associated with worse outcomes in patients receiving ICIs for advanced-stage NSCLC.

## 2. Materials and Methods

### 2.1. Patients

A single-center, retrospective cohort study of patients with a diagnosis of NSCLC treated at the University of Virginia (UVA) Health Cancer Center between 1 February 2015 and 1 January 2020 was performed with UVA IRB approval. Patients over the age of 18 with metastatic NSCLC treated with ICIs were included in the analysis. Two-hundred and nine (209) patients met the inclusion criteria. Patient demographics, smoking history, diagnostic imaging, laboratory testing, PD-L1 status, treatments received, duration of therapy (DOT), opioid prescriptions, and clinical outcomes were obtained and reviewed from the electronic medical record in accordance with UVA Institutional Review Board Guidelines. DOT was defined as the time from the first dose of ICI until permanent treatment discontinuation, which has also been referred to as time to treatment discontinuation (TTD) in the literature. We used DOT as a surrogate endpoint for progression-free survival due to the retrospective nature of this study and inconsistent imaging intervals. Furthermore, this method accounts for treatment beyond progression. DOT appears to correlate with PFS in NSCLC patients treated with ICI and both PFS and DOT correlate to a similar degree to OS [21].

### 2.2. Opioid Treatment and Assessment

Opioid utilization was determined in our cohort using the electronic medical records of all opioid prescriptions (fentanyl, hydrocodone, hydromorphone, methadone, morphine, and oxycodone) written while the patient received ICI therapy, including 2 weeks prior to ICI initiation, until permanent discontinuation. The total dose of opioids prescribed during the ICI treatment period was determined by the sum total of all prescriptions, calculated by multiplying the dose (mg) of opioid by the number of pills in each prescription. This total dose of opioids was converted to the morphine equivalent daily dose (MEDD) using established conversion factors (see Table 1). We defined high opioid use as an MEDD > 50, based on CDC guidelines for identifying patients with high opioid use [22]. Patients with high opioid use would exceed a hypothetical prescription of 5 mg of oral oxycodone every four hours, which has an approximate MEDD of 45, if prescribed continuously throughout the course of ICI treatment. We initially divided patients into two groups based on their degree of opioid use: high opioid use (MEDD ≥ 50) and low/no opioid use (MEDD < 50). After an initial review of the planned analysis, we subsequently divided the low/no opioid group into two groups: low opioid use (5 < MEDD < 50) and no/minimal opioid use (MEDD < 5) and performed statistical analyses to evaluate the relationship between opioid use and outcomes.

### 2.3. Statistical Analysis

Statistical analysis was performed using SPSS Version 28 for Windows (IBM Corp, Armonk, NY, USA). Duration of therapy (DOT) and overall survival were the co-primary end points of the analysis. DOT was defined as the time between the first dose of ICI until permanent treatment discontinuation. Overall survival (OS) was defined as the time between the first dose of ICI until date of death (patients alive at the time of data collection or lost to follow-up were censored at the date of last follow-up). DOT and OS were assessed using Kaplan–Meier and Cox regression methods. Independent sample t-tests and chi-square analysis were used for univariate comparisons.

A multivariate regression model was used to determine significant predictors of DOT and OS. Categorical variables in the multivariate regression model were adjusted for the following factors: age (continuous variable), gender, histology (squamous vs. non-squamous), Eastern Cooperative Oncology Group performance status (ECOG PS), PD-L1 status (>50% vs. <50%, if known), line of therapy (second or first line), smoking history (≥10 pack years vs. <10 pack year), opioid use (MEDD ≥ 50 vs. MEDD < 50), BMI (≥30 vs. <30), bone metastasis (no vs. yes), and tumor burden (≥2 sites of disease vs. <2 sites).

A separate model defining opioid use in three categories (high, low, and minimal/none) was performed to further elucidate the relationship between opioid use and outcomes. These outcomes were compared via Kaplan–Meier and Cox regression methods, though were not included in the univariate or multivariate analyses. Hazard ratios were calculated with their respective 95% confidence intervals.

For all comparisons and regressions, statistical significance was assigned at the *p* < 0.05 level.

## 3. Results

### 3.1. Patient Characteristics

Baseline patient characteristics are shown in Table 2. In this cohort study, the majority of patients were male (55%), ECOG PS of 0–1 (73%), and had adenocarcinoma tumor histology (69%). Thirty-seven patients (17.7%) were classified as high opioid users.

Patients with high opioid use were younger, had an ECOG PS of 2 or more, and a lower BMI. There were no statistically significant differences in gender, smoking history, tumor burden, PD-L1 status, line of therapy, or tumor histology among the three groups divided by opioid utilization.

### 3.2. Duration of Therapy and Overall Survival

**Figure 1:**
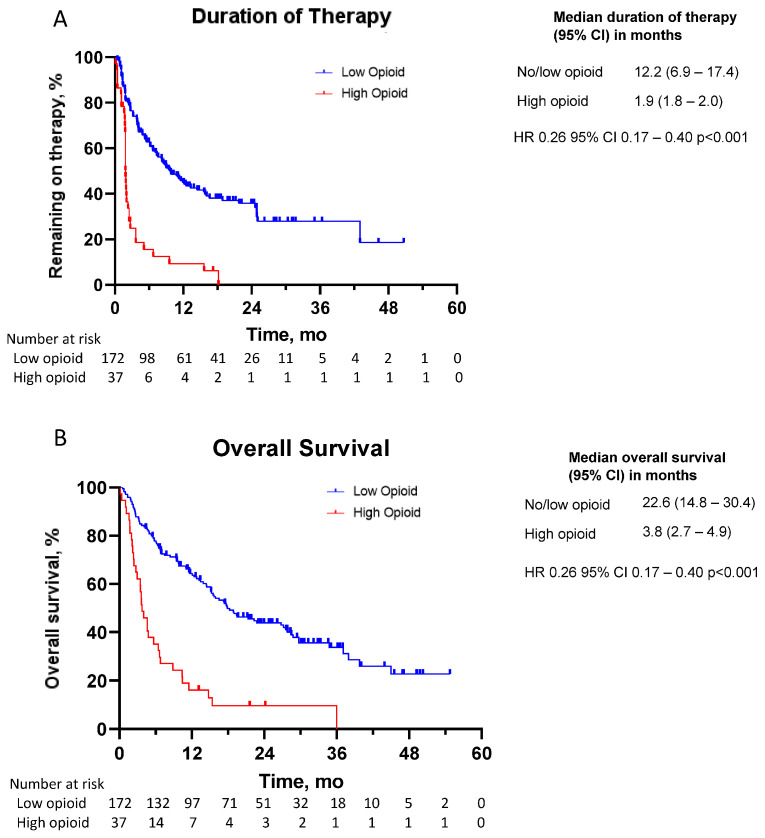
Kaplan–Meier curves for (**A**) duration of therapy and (**B**) overall survival in patients stratified by opioid use. Patients were stratified by no or low opioid use (MEDD < 50) or high opioid use (MEDD > 50). Censored data are indicated by tick marks. The number at risk includes the remaining number of patients included in the Kaplan–Meier analysis, shown at 6-month intervals.

In the initial analysis, in which patients were divided into two groups by opioid utilization, patients with no or low opioid use had a median OS of 22.6 months (95% CI: 14.8–30.4) compared to 3.8 months (95% CI: 2.7–4.9) for those with high opioid use (HR 0.26 95% CI: 0.17–0.40 *p* < 0.001, see Figure 1). Patients with no or low opioid use had a median DOT of 12.2 months (95% CI: 6.9–17.4) compared to 1.9 months (95% CI: 1.8–2.0) for those with high opioid use (HR 0.26 95% CI: 0.17–0.40; see Figure 1).

**Figure 2:**
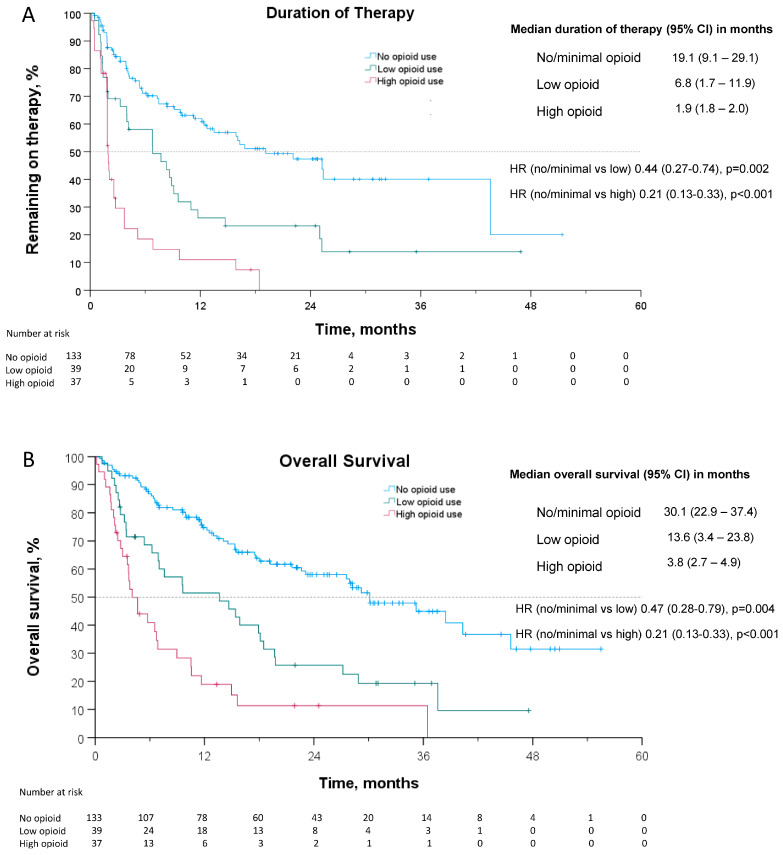
Kaplan–Meier curves for (**A**) duration of therapy and (**B**) overall survival. Given the results of the pre-planned analysis, patients were further stratified into 3 groups: no/minimal opioid Use (MEDD < 5), low opioid use (5 < MEDD < 50), and high opioid use (MEDD > 50). Censored data are indicated by tick marks. The number at risk includes the remaining number of patients included in the Kaplan–Meier analysis, shown at 6-month intervals.

In the subsequent analysis in which patients were divided into three groups by opioid utilization, patients with no/minimal, low, and high opioid use had a median DOT of 19.1 months (95% CI: 9.1–29.1), 6.8 months (95% CI: 1.7–11.9), and 1.9 months (95% CI: 1.8–2.0), respectively (see Figure 2). The hazard ratio for cessation of therapy or death (DOT) for no/minimal opioid use to low opioid use was 0.44 (95% CI: 0.27–0.74, *p* = 0.002) and for no/minimal opioid use to high opioid use was 0.21 (95% CI: 0.13–0.33, *p* < 0.001).

Patients with no/minimal opioid use, low opioid use, and high opioid use had a median OS of 30.1 months (95% CI: 22.9–37.4), 13.6 months (95% CI: 3.4–23.8), and 3.8 months (95% CI: 2.7–4.9), respectively (see Figure 2). The hazard ratio for death for no/minimal opioid use compared to low opioid use was 0.47 (95% CI: 0.28–0.79, *p* = 0.004) and for no/minimal opioid use compared to high opioid use was 0.21 (95% CI: 0.13–0.33, *p* < 0.001).

### 3.3. Univariate and Multivariate Analyses

**Figure 3:**
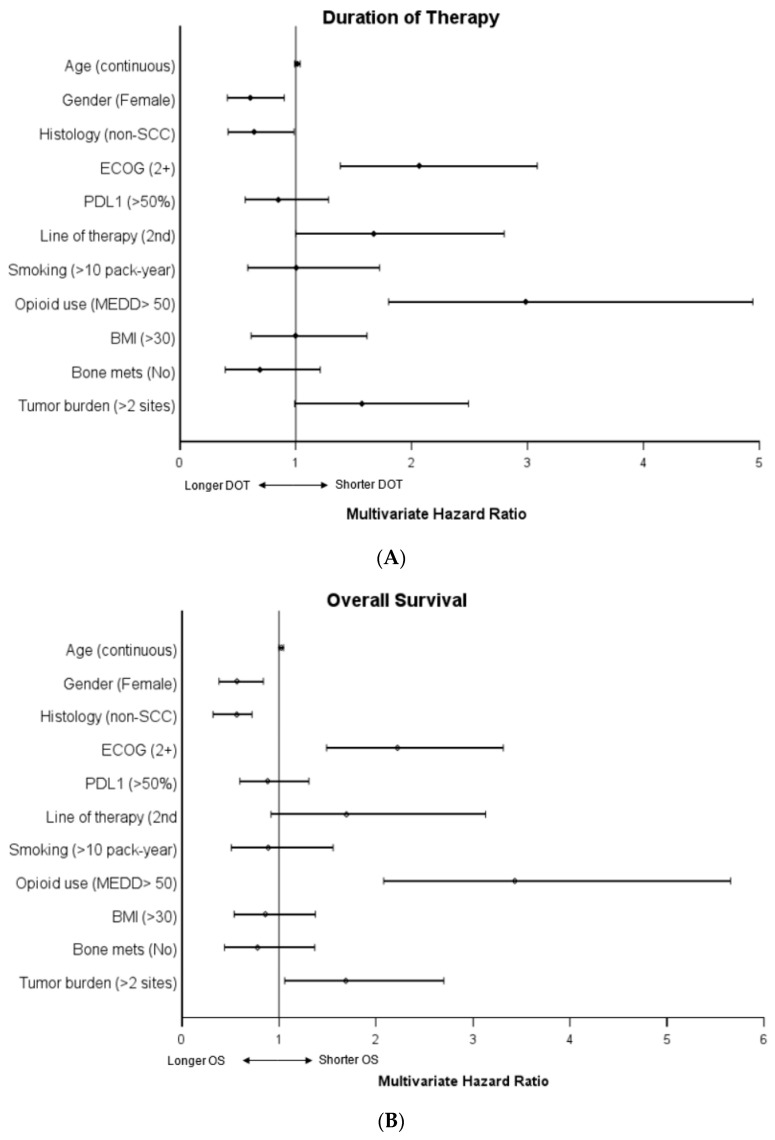
Multivariate COX regression hazard ratios of (**A**) duration of therapy and (**B**) overall survival. For each category, the designation listed in parentheses was compared to the opposite reference variable. Age was tested as a continuous variable. For example, the hazard ratio of death (OS) in the category female gender was compared to the reference variable male gender. Reference variables by category: age (N/A, continuous), gender (male), ECOG (0–1), line of therapy (first line), smoking (never or <10 pack-years), MEDD group (low, MEDD < 50), BMI < 30, bone mets (yes), tumor burden (<2 sites of disease).

Male gender, squamous histology, ECOG PS > 2, later line therapy, and high opioid use were significantly associated with a shorter duration of therapy in the univariate analysis. Line of therapy was not significant in the multivariate analysis, while the other factors remained significant. There was no significant association with age, PD-L1 status, smoking status, BMI, bone metastasis or tumor burden, and duration of therapy in the univariate or multivariate analyses (see Table 3 and Figure 3).

Male gender, squamous histology, ECOG PS > 2, and high MEDD were significantly associated with decreased overall survival on univariate analysis. These remained significant in the multivariate analysis. While not significant in the univariate analysis, increased tumor burden was associated with worse overall survival in the multivariate analysis. There was no significant association with age, PD-L1 status, line of therapy, smoking status, BMI, bone metastasis, and overall survival in the univariate or multivariate analyses (see Table 3 and Figure 3)

## 4. Discussion

This retrospective analysis of patients with metastatic NSCLC receiving ICIs demonstrated that high opioid use, defined as a MEDD > 50, is associated with reduced OS and a shorter DOT in patients with advanced NSCLC receiving an ICI. The effect was dramatic and significant even when accounting for possible confounding variables including age, ECOG PS, PD-L1 status, line of therapy, bone metastasis, and tumor burden in the multivariate analysis.

Patients with high opioid use were more likely to have a lower BMI and have a poor ECOG PS, which have portended worse outcomes in patients with NSCLC. In the multivariate analysis, line of therapy did not meet our cutoff of statistical significance for independently impacting duration of therapy or overall survival. ECOG status > 2, tumor burden (>2 sites of disease), and high opioid use were independently and statistically associated with a worse DOT and OS, whereas female gender and non-squamous histology were associated with longer a DOT and OS. The hazard ratios for cessation of therapy or death (DOT) and death (OS), both 0.26, among low opioid users as compared to high opioid users were clinically significant.

There are emerging data that support our findings that concomitant opioid use in patients receiving ICIs may negatively impact outcomes in patients with NSCLC. Taniguchi et al. reported that patients receiving both opioids and nivolumab had a significantly lower overall response rate (ORR) with shorter PFS and OS compared to a case-matched cohort of patients receiving only nivolumab, with a 5 month difference in median overall survival [23]. In another study examining the use of antibiotics and other concomitant medications (including opioids) in NSCLC patients receiving ICIs, Iglesias-Santamaria and colleagues found that the use of opioids was associated with reduced PFS and OS in a cohort of 102 patients in a variety of advanced malignancies, including NSCLC, renal, bladder, melanoma, and head and neck [24]. Though not analyzed by tumor type, the difference in median survival (791 vs. 258 days) was similar to our study at about 17 months. In recent years, several other studies have shown similar findings to our results. Cotogni et al. performed a systematic review and identified 13 retrospective analyses associating worse outcomes with opioid use compared to no opioid use among cancer patients (largely melanoma and NSCLC) treated with ICIs [25]. Guo et al. performed a systemic review and meta-analysis of published reports—largely in melanoma and NSCLC—evaluating outcomes in patients treated with ICIs, which included 6 retrospective studies, and also evaluated studies assessing the association of NSAIDs with outcomes [26]. Their meta-analysis, utilizing a fixed-effects inverse-variance model, showed that opioid use compared to no opioid use was associated with lower response rates, as well as lower PFS and OS. A statistically significant association was not seen when comparing patients using NSAIDs vs. no NSAIDs.

It is important to note that opioid use in general has been associated with worse outcomes in NSCLC, prior to the use of ICIs. Prolonged and chronic opioid use was associated with reduced survival in NSCLC patients who underwent curative resection [27]. Any opioid use over five daily morphine equivalents (MEDD) in patients with advanced NSCLC has been associated with worse overall survival [28]. Pre-clinical data and animal models provide insight into potential explanations for these worse outcomes noted clinically. Metastatic NSCLC tumors exhibit increased mu-opioid receptor expression relative to adjacent lung tissue or normal controls [29]. Opioids directly stimulate tumor growth and progression via mu-opioid receptors and costimulation of the epidermal growth factor receptor (EGFR) [29,30]. Opioids also promote angiogenesis, lead to epithelial–mesenchymal transition, and promote metastasis [31]. These effects can be blocked by the mu-opioid receptor antagonist methylnaltrexone [31]. In an NSCLC xenograft model, mu-opioid receptor overexpression led to tumor growth and metastasis in nude mice, in which the thymus is absent, suggesting that these effects are mediated through the mu-opioid receptor as opposed to the known immunosuppressive effects of opioids [32]. While there are many studies that show that opioids promote tumor growth, angiogenesis, and metastasis, there are some conflicting data with other studies showing that opioids increase cell cycle arrest, apoptosis, and have anti-angiogenic properties [33].

Despite consistent reports that opioid use is associated with worse survival in NSCLC, Hasegawa et al. [34] found no difference in OS when patients using opioids were stratified by high (>60 MEDD) vs. low (<60 MEDD) opioid use, with essentially overlapping Kaplan–Meier survival curves. This study examined patients prior to the widespread incorporation of ICIs into clinical practice. Given this report, we sought to evaluate the impact of high opioid use compared to low opioid use on clinical outcomes among patients treated with ICIs. A dose-dependent correlation with outcomes (high opioid use associated with even worse outcomes than low opioid use) could indicate an interaction between opioid use and ICIs. We analyzed DOT and OS outcomes in our cohort among high opioid vs. low opioid vs. no/minimal opioid users. We included patients with an MEDD of <5 in the no/minimal opioid group, as this level of opioid use has been found to distinguish patients with intermittent/occasional opioid use from those with ongoing scheduled use [35]. In addition, while opioid use has been associated with worse outcomes in NSCLC, this was not seen in patients with an MEDD of 0–5 [28]. In contrast to Hasegawa et al., we found that high opioid use (MEDD > 50) was associated with decreased OS as compared to low opioid use (5 < MEDD < 50), and patients with low opioid use had decreased OS compared to those with no/minimal opioid use. Weinfeld et al. performed a similar retrospective analysis of 212 cancer patients treated with ICIs and found a similar association of worsening outcomes with lower and higher doses of opioids compared to no opioid use (high opioid use defined as an MEDD > 60) [25]. As noted above, Taniguchi et al. found a significant difference not only in PFS and OS with opioid use in advanced NSCLC treated with nivolumab, but also in the response rate (ORR 21.1% vs. 2.6%) [23]. In addition to these reports, several additional retrospective analyses have found reduced response rates, PFS, and OS associated with opioid use in patients receiving ICIs, which were recently summarized in two separate systematic reviews [26,36].

Taken together, the dose-dependent association of worse outcomes with higher opioid use seen in our analysis (not previously seen in the pre-immunotherapy era) and the decreased response rate to ICIs among those with opioid use support the possibility of a mechanistic interaction between opioids and ICI therapy. Potential mechanisms by which opioid use could impact ICI efficacy include the alteration of the immune response via the attenuation of T-cell activity or the alteration of the gut microbiome. Acute and chronic exposure to morphine suppresses CD8+ T-cell activity [37]. Opioid use increases the number of Tregs, which are thought to repress antitumor immunity, and tumor-infiltrating Tregs have been associated with a worse prognosis in NSCLC [13,38]. Additionally, opioids influence the gut microbiome, which may modulate the complex interplay between the cancer immune response and the gut microbiome [16], as alterations of the microbiome have been associated with worse outcomes among patients receiving ICIs [17,18]. While the effects of opioids on tumor growth, progression, and metastasis via the mu-opioid receptor may be independent of the immune system [32], the effects of opioids on the immune system may play a role in patients receiving ICIs, and could explain why higher opioid use in our cohort was associated with even worse outcomes. The growing evidence associating worse response rates and survival with concomitant opioid use and ICI therapy warrants further investigation into a mechanistic interaction between opioids and ICI therapy.

The reason for the association of worse survival in NSCLC among patients with opioid use cannot be elucidated in a retrospective review. While the effects of opioids via the mu-opioid receptor may directly impact outcomes, the interactions between opioids, the immune system, and ICIs may occur through a complex interplay of mechanisms. Pain (and thus opioid use) itself may be associated with other features expected to portend a poor prognosis, including more aggressive disease. Pain, independent of opioid use, has also been associated with worse outcomes in NSCLC patients [28]. While we have tried to control for features that may be associated with a worse prognosis, such as ECOG PS, disease burden and bone metastasis, our and others’ data are limited by the inherent limits of retrospective studies.

Prospective studies showing improved outcomes with opioid-sparing strategies would further support a causative relationship of opioids and poor outcomes, whatever the mechanism. These studies are difficult to perform, as opioids have become a cornerstone of treating cancer-related pain and care must be taken with such trials to not undertreat pain. Despite this, there are some prospective data to support limiting systemic opioid use or blunting the systemic effect of opioids in cancer patients. Patients with metastatic cancer randomized to implantable intrathecal drug delivery systems compared to standard medical management saw a significant reduction in their systemic opioid use (290 MEDD vs. 50 MEDD), which led to improved pain scores, decreased toxicity from systemic opioids, and improved survival at 6 months [39]. In an unplanned post hoc analysis of two randomized trials assessing the use of methylnaltrexone in opioid-induced constipation, cancer patients who received methylnaltrexone had improved overall survival compared to those who received the placebo, a difference not seen in non-cancer patients [40]. To prospectively validate our findings, we suggest prospective trials comparing an opioid-sparing pain management strategy with standard management among patients receiving ICIs. This approach would allow for an analysis of the impact of opioid use on outcomes without the inherent limitations of the retrospective studies performed to date. Without prospective data, the clinical applicability of our data remains limited, as opioids remain the standard of care in managing cancer-related pain.

This study has several limitations. This is a single-center, retrospective study, with inherent limitations. There may be independent variables affecting outcomes not accounted for in our multivariate model. In addition, opioid prescription data were only available for review if provided within our institution due to the statutory limitations associated with the review of patient-specific statewide prescription monitoring. It is possible that additional prescriptions were provided to patients in both cohorts without our knowledge, or that patients took fewer opioids than prescribed. This study only analyzed opioids prescribed to calculate an MEDD, but the exact quantity of opioids utilized by patients is unknown. For patients receiving recurring prescriptions, which would include all of those falling into the high use category, it is likely that most were taking their full prescription on a regular basis as prescribed. Pain has been associated with worse outcomes in advanced NSCLC, and we were unable assess the impact of non-opioid analgesics in our study. Comparing outcomes among patients with opioid use by indication (i.e., cancer-related vs. not) would have made our analysis more robust, but we were unable to reliably identify patients who had chronic opioid exposure prior to treatment, as the majority of our patients were not a part of our system prior to their NSCLC diagnosis.

## 5. Conclusions

High opioid use among patients with advanced NSCLC receiving ICIs was independently associated with reduced OS and a shorter DOT when compared to those with low or no opioid use. Our findings contribute to a growing body of evidence that concurrent opioid use is associated with worse outcomes among NSCLC patients treated with immunotherapy. These results should encourage providers to use opioids judiciously in NSCLC patients receiving ICIs when treating cancer-related pain and maximize, where possible, the use of opioid-sparing pain management strategies. The development of opioid-sparing symptom management, the role of peripheral opioid antagonists, and the influence on clinical outcomes warrant further investigation. Additionally, while our study examined outcomes in NSCLC patients, additional investigations examining the impact of opioids on response to ICIs should be considered in malignancies where ICIs are commonly used. Our study should prompt further investigation into the biologic mechanisms by which opioids suppress immune responses among patients receiving ICIs.

## Figures and Tables

**Table 1:** Morphine equivalent daily dose (MEDD) calculation.

	Conversion Factor	MED (Morphine Equivalent Dose)
Fentanyl	2.4	MED = Total dose of opioid prescribed during ICI treatment × conversion factor
Hydrocodone	1	
Hydromorphone	4	
Methadone	10	MEDD (morphine equivalent daily dose)
Morphine	1	MEDD = MED/total # days during ICI treatment
Oxycodone	1.5	

The total dose of opioids prescribed during the ICI treatment period (defined as 2 weeks prior to initiation of treatment until permanent discontinuation) was calculated by multiplying the dose (mg) of opioid by the number of pills in each prescription. This total dose of opioids was converted to morphine equivalents by multiplying the specific opioid used by the corresponding morphine equivalent dose (MED) conversion factor. The conversion factor for each opioid is listed in the table. To calculate the morphine equivalent daily dose (MEDD), MED was divided by the total number of days in the ICI treatment period.

**Table 2:** Patient characteristics.

	No/Minimal Opioid Use (Average MEDD < 5)	Low Opioid Use (Average MEDD 5–50)	High Opioid Use (Average MEDD > 50.0)	*p*-Value
N	133	39	37	
Median age (range)	67 (37–92)	59 (46–82)	59 (42–76)	0.001
Gender (percentage)				0.404
Male	71 (53.4%)	20 (51.3%)	24 (64.9%)	
Female	62 (46.6%)	19 (48.7%)	13 (35.1%)	
Histology (percentage)				0.115
Adenocarcinoma	100 (75.2%)	24 (61.6%)	21 (56.8%)	
Squamous	23 (17.3%)	10 (25.6%)	14 (37.8%)	
Other/unknown	10 (7.5%)	5 (12.8%)	2 (5.4%)	
Performance status (ECOG)				0.001
0–1	108 (81.2%)	25 (64.1%)	19 (51.4%)	
2+	25 (18.8%)	14 (35.9%)	18 (48.6%)	
Tumor burden				0.894
≤2 Sites of metastases	88 (66.2%)	25 (64.1%)	23 (62.2%)	
>2 Sites of metastases	45 (33.8%)	14 (35.9%)	14 (37.8%)	
BMI				0.016
<30	96 (72.2%)	29 (74.4%)	35 (94.6%)	
≥30	37 (27.8%)	10 (25.6%)	2 (5.4%)	
PD-L1				0.225
≥50%	44 (33.1%)	15 (38.4%)	6 (16.2%)	
1–49%	22 (16.5%)	7 (18.0%)	6 (16.2%)	
0%	26 (19.6%)	10 (25.6%)	9 (24.3%)	
Unknown	41 (30.8%)	7 (18.0%)	16 (43.3%)	
Treatment				0.181
ICI alone (frontline)	36 (27.1%)	10 (25.6%)	4 (10.8%)	
ICI (2nd line or further)	50 (37.6%)	18 (46.2%)	21 (56.8%)	
Chemo + ICI	47 (35.3%)	11 (28.2%)	12 (32.4%)	
Smoking history				0.832
Never/<10 pack years	32 (24.1%)	4 (10.3%)	7 (18.9%)	
10–50 pack years	22 (16.5%)	25 (64.1%)	20 (54.1%)	
>50 pack years	79 (59.4%)	10 (25.6%)	10 (27.0%)	
Genomics ^1^				NC ^2^
EGFR	0 (0%)	1 (2.6%)	0 (0%)	
ROS	0 (0%)	1 (2.6%)	0 (0%)	
BRAF	2 (1.5%)	1 (2.6%)	0 (0%)	
KRAS	13 (9.8%)	1 (2.6%)	2 (5.4%)	

Patient characteristics. Abbreviations: ECOG: Eastern Cooperative Oncology Group; BMI: body mass index; PD-L1: programmed death ligand 1; ICI: checkpoint inhibitor. ^1^ No patients were identified as having ALK, MET, RET, or NTRK alterations. ^2^ Not calculated; number too small for meaningful comparisons to be made.

**Table 3:** Univariate and multivariate hazard ratios.

(A) Duration of Therapy				
	Univariate HR	*p*-Value	Multivariate HR	*p*-Value
Age (continuous)	1.01 (0.99–1.03)	0.616	1.01 (0.99–1.04)	0.288
Gender (female vs. male)	0.62 (0.42–0.90)	0.012	0.61 (0.41–0.90)	0.013
Histology (non-squamous vs. squamous)	0.49 (0.33–0.73)	0.0001	0.64 (0.42–0.99)	0.043
ECOG (2+ vs. 0–2)	2.37 (1.64–3.44)	0.0001	2.07 (1.38–3.08)	0.0001
PDL1 (50% or higher vs. <50%)	0.70 (0.48–1.01)	0.057	0.85 (0.56–1.28)	0.437
Line of therapy (second vs. first line)	1.85 (1.14–2.99)	0.013	1.67 (0.99–2.80)	0.051
Smoking (≥10 pack-year vs. <10)	0.99 (0.59–1.66)	0.970	1.00 (0.58–1.72)	0.989
Opioid use (MEDD ≥ 50 vs. <50)	3.87 (2.52–5.95)	0.0001	2.98 (1.80–4.94)	0.0001
BMI (30 or above vs. <30)	0.75 (0.49–1.15)	0.186	1.00 (0.62–1.61)	0.989
Bone metastasis (no vs. yes)	1.08 (0.69–1.70)	0.738	0.69 (0.39–1.21)	0.193
Tumor burden (≥2 sites of disease vs. <2)	1.15 (0.78–1.70)	0.484	1.57 (0.99–2.49)	0.053
**(B) Overall Survival**				
	**Univariate HR**	***p*** **-Value**	**Multivariate HR**	***p*** **-Value**
Age (continuous)	1.01 (0.99–1.03)	0.504	1.02 (1.00–1.05)	0.050
Gender (female vs. male)	0.54 (0.37–0.79)	0.001	0.57 (0.38–0.84)	0.005
Histology (non-squamous vs. squamous)	0.51 (0.34–0.74)	0.001	0.57 (0.32–0.72)	0.001
ECOG (2+ vs. 0–2)	2.76 (1.90–4.02)	0.0001	2.22 (1.49–3.31)	0.0001
PDL1 (50% or higher vs. <50%)	0.76 (0.52–1.10)	0.143	0.89 (0.60–1.31)	0.597
Line of therapy (second vs. first line)	1.53 (0.94–2.48)	0.085	1.70 (0.92–3.13)	0.091
Smoking (≥10 pack-year vs. <10)	0.92 (0.55–1.54)	0.749	0.89 (0.51–1.56)	0.686
Opioid use (MEDD ≥ 50 vs. <50)	3.84 (2.51–5.87)	0.0001	3.43 (2.08–5.66)	0.0001
BMI (≥30 vs. <30)	0.69 (0.45–1.05)	0.083	0.86 (0.54–1.38)	0.533
Bone metastasis (no vs. yes)	1.19 (0.76–1.86)	0.456	0.78 (0.44–1.37)	0.384
Tumor burden (≥2 sites of disease vs. <2)	1.21 (0.83–1.77)	0.330	1.69 (1.06–2.70)	0.029

Univariate and multivariate Cox proportional hazard models were used to generate hazard ratios which estimate the risk of (**A**) cessation of therapy or death (duration of therapy) and (**B**) the risk of death (overall survival). Each hazard ratio compares the risk of the first category—listed in parentheses—to the second, except for age, which was performed as a continuous variable. The 95% confidence intervals for each hazard ratio are listed in parentheses.

## Data Availability

The data presented in this study are available on request from the corresponding author.
